# Case of Vesuo-Vaginal Fistula—Operation on

**Published:** 1866-07

**Authors:** Darius Mason

**Affiliations:** Prairie du Chien, Wis.


ARTICLE XXIV.

### CASE OF VESUO-VAGINAL FISTULA—OPERATION ON.

By DARIUS MASON, M.D., Prairie du Chien, Wis.

Jan. 7th, 1862. I was called to see Mrs. W., aged 18 years, married about 12 months. She had been delivered of a male child, weighing 10 pounds, on the 1st of November, 1861, after a severe labor of about 60 hours. She was apparently convalescing well, until the 6th of November, when she discovered her urine passing involuntarily, and which had so continued up to the time of my visiting her. Upon examination by passing an ordinary female catheter into the bladder, the tip of the forefinger, *per vaginam*, came in direct contact with the catheter, through an oval opening about five lines across its long diameter transversely across the vaginal wall, and about two inches from the orifice of the urethra. The patient being in other respects in good health, I proposed an operation as soon as convenient.

Assisted by the late Dr. B. F. White, of Prairie du Chien, and Drs. Andros and Martin, of McGregor, Iowa, I operated on the 17th of January. The patient put under chloroform, and placed in lithotomy position, and by the aid of Dr. Simm's retractor, or duck-bill speculum, the fistulous opening was brought into view, when, with a long-handled knife, the vaginal mucous membrane was removed completely round the opening, extending back from its edge about two and a-half lines. I next passed four silver wire sutures, about 12 inches long, by a needle held by the *porte-aiguille* piercing the mucous membrane about two lines from the cut margin, and making its exit at the margin of the opening, and entering the opposite margin of the opening and again making its exit through the mucous membrane, the same distance from the cut surface as at the first point of entrance; a perforated silver plate or button, of an oval shape, slightly concavo-convex, about three-fourths of an inch long, was next passed down upon the wound, the two ends of each suture passing through a perforation in the button or plate; the next step, was the passing of a perforated shot over each suture snugly down upon the plate and firmly pinched by a strong forceps, the wires were then cut close to the shot. The patient was then placed in bed upon her back, a sigmoid catheter introduced with a flexible tube attached, a single grain of opium was given immediately, and one grain every four hours for the next forty-eight hours; diet, dry toast and tea. The catheter was removed and cleansed every morning.

On the 19th, menstruation commenced, and continued through the 21st, after which, the vagina was washed every morning with tepid water. All the urine passed by the catheter, which produced very little irritation until the 24th, when it began to be very troublesome, and it was removed for two hours at a time during the day.

On the 26th, assisted by the late Dr. WHITE, I removed the plate, and found the union perfect, except a small spot about the centre of the wound which appeared to be a little ulcerated; all the urine passed by catheter introduced every four hours. By the aid of a warm water enema, the bowels were moved on the 27th. On the 29th, a little urine escaped involuntarily, and examination revealed a small opening through which an ordinary director might be passed, at the point of apparent ulceration mentioned, and through which the urine was escaping.

It was thought best that the patient should rest a little from this operation before undergoing another; consequently, the second operation was deferred until the 20th of February, when the same steps were gone through with as in the first operation, the sutures and plate being removed on the 20th of February, and the wound was well united, and, I may say, she has had good health up to the present time, having no difficulty in retaining or voiding urine.

On the 4th of October, 1865, she was delivered of a female child, weighing about seven pounds, after an easy labor of about four hours.

